# Physics-Informed Neural Networks Integrating Compartmental Model for Analyzing COVID-19 Transmission Dynamics

**DOI:** 10.3390/v15081749

**Published:** 2023-08-16

**Authors:** Xiao Ning, Jinxing Guan, Xi-An Li, Yongyue Wei, Feng Chen

**Affiliations:** 1 State Key Laboratory of Bioelectronics, School of Biological Science and Medical Engineering, Southeast University, 2 Sipailou, Nanjing 210096, China; 2 Center for Global Health, Departments of Epidemiology and Biostatistics, School of Public Health, Nanjing Medical University, Nanjing 211166, China; 3 Ceyear Technology Co., Ltd., 98 Xiangjiang Road, Qingdao 266000, China; 4 Public Health and Epidemic Preparedness and Response Center, Peking University, Xueyuan Road, Haidian District, Beijing 100191, China

**Keywords:** compartmental models, forward-inverse problem, physics-informed neural networks, COVID-19 transmission

## Abstract

Modelling and predicting the behaviour of infectious diseases is essential for early warning and evaluating the most effective interventions to prevent significant harm. Compartmental models produce a system of ordinary differential equations (ODEs) that are renowned for simulating the transmission dynamics of infectious diseases. However, the parameters in compartmental models are often unknown, and they can even change over time in the real world, making them difficult to determine. This study proposes an advanced artificial intelligence approach based on physics-informed neural networks (PINNs) to estimate time-varying parameters from given data for the compartmental model. Our proposed PINNs method captures the complex dynamics of COVID-19 by integrating a modified Susceptible-Exposed-Infectious-Recovered-Death (SEIRD) compartmental model with deep neural networks. Specifically, we modelled the system of ODEs as one network and the time-varying parameters as another network to address significant unknown parameters and limited data. Such structure of the PINNs method is in line with the prior epidemiological correlations and comprises the mismatch between available data and network output and the residual of ODEs. The experimental findings on real-world reported data data have demonstrated that our method robustly and accurately learns the dynamics and forecasts future states. Moreover, as more data becomes available, our proposed PINNs method can be successfully extended to other regions and infectious diseases.

## 1. Introduction

Modelling and predicting the behaviour of infectious diseases is crucial for early warning and evaluating effective interventions to mitigate damage. However, since real-world data can be inherently stochastic, noisy, and even inaccessible, model optimization and methodological innovation are urgently needed to handle imperfect data and provide early warning of major public health emergencies [1]. Epidemic compartmental models, governed by a nonlinear system of ordinary differential equations (ODEs), simulate multi-state population transitions to characterize the transmission dynamics of infectious diseases [2]. These models provide the flexibility to incorporate additional compartments or assumed parameters, facilitating the exploration and estimation of the impact of various interventions. The parameters included in the compartmental model, determine the transmission progress between different disease statuses and can generate essential characteristics of an epidemic [3]. However, compartmental models coupled with diverse physical and biological processes are complex with many unknown parameters, posing significant challenges in the realm of parameter estimation from available data, this process is referred to as the forward-inverse problem (inverse problem). Many research efforts focus on parameter estimation of epidemiological models, which involves converting the inverse problem into an optimization problem and formulating an estimator by minimizing an objective function [4,5,6]. However, these methods encounter noteworthy limitations that impede their practical applications. One limitation is the exponential increase in computational cost for numerical simulations as the complexity and models. Additionally, these parameter estimation methods are only applicable to time-constant parameters, failing to capture the complex dynamics of infectious diseases over time in real-world scenarios [7,8].

Epidemic compartmental models play a critical role in detecting, understanding, and combating infectious disease outbreaks and have been widely used to fight against the COVID-19 pandemic. However, since various non-pharmaceutical interventions (NPIs) are employed during the evolution of COVID-19, some model parameters are time-varying. Wang et al. divided the dynamics of COVID-19 in Wuhan from 1 January to 8 March 2020, into five time periods and used Markov Chain Monte Carlo (MCMC) method to estimate the parameters for each period, demonstrating the high covert and high transmissibility characteristics of the outbreak [9]. Identifying time-varying parameters in compartmental models is a complex inverse problem, making it challenging to accurately model outbreak dynamics [10,11]. Recent advances in Physics-informed neural networks (PINNs) have shown promise in various fields by incorporating prior knowledge into deep neural networks (DNNs) to enhance their accuracy and robustness [12,13], and have gained considerable attention in various domains [14,15]. These methods have proven effective in solving partial differential equations (PDEs) and can be integrated into epidemic compartmental models to model complex outbreak dynamics, particularly with respect to COVID-19 [16,17,18,19,20]. For example, Kharazmi et al. used PINNs to identify time-varying parameters and data-driven fractional differential operators in several epidemiological models [18]. Long et al. proposed a variant of PINNs to fit daily reported cases and identify time-varying parameters in the susceptible-infectious-recovered-deceased (SIRD) model for the spread of COVID-19 [19]. Nascimento et al. introduced an approach that combines physics-informed and data-driven kernels to reduce the gap between predictions and observations [21]. Cai et al. employed fractional PINNs to refine the classical susceptible–exposed–infected–removed (SEIR) model, infer time-varying parameters, and identify unobserved dynamics of the fractional SEIR model [20]. These advancements offer promising tools to enhance the understanding of infectious disease outbreaks, significantly contributing not only to the response to the ongoing COVID-19 pandemic but also to the potential application in other infectious diseases [22].

PINNs bring the advantage of incorporating domain knowledge and physical laws into neural network architectures, making them well-suited for solving inverse problems in epidemiological modelling. However, most of these approaches only consider the transmission rate as a function of time, while setting other parameters to fixed values. Additionally, they mainly use time-varying parameters for prediction and lack a systematic epidemiological analysis. The primary focus of this paper lies not only in introducing the PINNs method for estimating time-varying parameters in the compartmental model and performing future predictions but also in analyzing the impact of non-pharmaceutical interventions (NPIs) based on the estimated parameters. To model the dynamics of COVID-19, we modified the classical Susceptible-Infectious-Removed (SIR) model by introducing additional compartments and coefficients. We then tested the effectiveness of proposed method using real-world reported data, simulation experiments showed that our proposed PINNs method effectively performs data-driven parameter estimation for modelling COVID-19 transmission. The estimated parameters were analyzed quantitatively and qualitatively in the context of the corresponding interventions and were found to be consistent with expected dynamics and previous research. Therefore, the primary contributions of this paper are as follows:

- We employed the PINNs method, which combines mathematical modelling and neural network modelling to efficiently address the complexity of infectious disease transmission dynamics in real-world scenarios. The proposed PINNs structure considers several coefficients of the epidemic compartmental model as time-varying parameters, which provides a more realistic representation and enables accurate capturing of transmission dynamics for reliable predictions.
- We constructed a SEIRD compartmental model that takes an incubation period and the corresponding infectivity into account, including both unknown time-varying and constant parameters. Given many unknown parameters and limited data, we modelled the system of ODEs as one network and the time-varying parameters as another network to reduce the parameter of neural networks. Furthermore, such structure of the PINNs method is in line with the prior epidemiological correlations.
- The experiment is conducted on real-world COVID-19 data to verify the effectiveness of our proposed PINNs method. Experiment results show that our proposed method provides accurate capture of COVID-19 dynamics and reliable predictions in Italy. Additionally, the effective reproduction number $R_{t}$ was calculated based on the time-varying compartmental model to analyze the dynamics of COVID-19. Moreover, as more data becomes available, it can be successfully extended to model and analyze infectious disease transmission dynamics in various regions and for different infectious diseases.

The rest of the paper is organized as follows: Section 2 presents the compartmental model and the proposed PINNs method, as well as their implementation details, are introduced. In Section 3, we present simulation results based on the early outbreak reported data in Italy. Then, in Section 4, we further present some discussions and suggestions. Finally, a brief conclusion is made in the last section.

## 2. Methodology

The PINNs framework refers to a data-driven method that approximates the solution of differential equations and provides estimates for unknown parameters. The main idea of PINNs is to integrate a priori knowledge as physical laws or domain expertise modelled by differential equations into deep neural networks.

### 2.1. Compartmental Model

The first SIR compartmental model was proposed by Kermack and McKendrick to study the epidemics of the Black Death in London and the plague in Mumbai [2]. These models are generally represented as the following nonlinear dynamical system:

(1) $$\begin{array}{c} \left\{\begin{array}{c} \begin{array}{cc} \frac{dU(t)}{dt} & =F(t,U(t);\Xi )\\ U(t_{0}) & =U_{0} \end{array} \end{array}\right. \end{array}$$

where $U\left(t\right)\in R^{D}$(typically D≫1) is the state variable, $t\in [t_{0},\;T]$ is the time range, $U(t_{0})$ is the initial state, and Ξ stands for the parameters of the dynamical system.

The SIR compartmental model provided the simplest framework that matched the reporting structure with the least underlying assumptions. Many variations of the SIR model have been proposed to analyze the transmission of COVID-19. In this paper, we consider a geographical region as isolated from other regions, and within such region we divide the population (*N*) of the study region into five compartments, susceptible (*S*, vulnerable to COVID-19 infection), exposed (*E*, latent individual or asymptomatic infective), infected (*I*, symptomatic infected), recovered (*R*, immune to COVID-19), and dead (*D*, death due to COVID-19). The details of the SEIRD model are described below:

(2) $$\begin{array}{c} \left\{\begin{array}{c} \begin{array}{cc} \frac{dS(t)}{dt} & =-\frac{\beta S(t)(\epsilon E(t))+I(t)}{N}\\ \frac{dE(t)}{dt} & =\frac{\beta S(t)(\epsilon E(t)+I(t))}{N}-\frac{E(t)}{\alpha }\\ \frac{dI(t)}{dt} & =\frac{E(t)}{\alpha }-\gamma I\left(t\right)-\mu I\left(t\right)\\ \frac{dR(t)}{dt} & =\gamma I(t)\\ \frac{dD(t)}{dt} & =\mu I(t)\\ N & =S(t)+E(t)+I(t)+R(t)+D(t) \end{array} \end{array}\right. \end{array}$$

where S(t), E(t), I(t), R(t), D(t) denote the number of susceptible, exposed, infectious, recovered, and deceased individuals over time respectively, along with non-negative initial conditions $S\left(0\right)=S_{0},\;E\left(0\right)=E_{0},\;I\left(0\right)=I_{0},\;R\left(0\right)=R_{0},\;D\left(0\right)=D_{0}$. β≥0 represents the transmission rate, which represents the probability of infection per exposure when a susceptible individual (*S*) has contact with an infected patient (*I*) and becomes a latent exposed individual (*E*). A coefficient parameter ϵ is introduced since the transmission capacity of exposed and onset populations may be different. ϵβ represents the potential rate per exposure when a susceptible individual (*S*) has mutual contact with an exposed individual (*E*), and transmits it to another exposed individual (*E*). α is the average duration of incubation period, 1/α is the rate of latent individuals becoming infectious. Besides, γ≥0 represents the recovery rate, μ≥0 represents the death rate, and *N* is the total population.

The assumption that the parameters in Equation (2) are time-constant, which is a highly restrictive and unrealistic one for the real-world epidemic where various interventions exist. The associated interventions implemented by authorities, and/or mutations of the virus, et al. make the compartmental model require time-varying parameters to capture the dynamic of COVID-19. Therefore, by considering transmission rate β, recovery rate γ and death rate μ as functions of time β(t), γ(t), μ(t), the re-written SEIRD model is as follows:

(3) $$\begin{array}{c} \left\{\begin{array}{c} \begin{array}{cc} \frac{dS(t)}{dt} & =-\frac{\beta (t)S(t)(\epsilon E(t))+I(t))}{N}\\ \frac{dE(t)}{dt} & =\frac{\beta (t)S(t)(\epsilon E(t))+I(t))}{N}-\frac{E(t)}{\alpha }\\ \frac{dI(t)}{dt} & =\frac{E(t)}{\alpha }-\gamma \left(t\right)I\left(t\right)-\mu \left(t\right)I\left(t\right)\\ \frac{dR(t)}{dt} & =\gamma (t)I(t)\\ \frac{dD(t)}{dt} & =\mu (t)I(t)\\ N & =S(t)+E(t)+I(t)+R(t)+D(t) \end{array} \end{array}\right. \end{array}$$

Among them, the five variables S(t), E(t), I(t), R(t), D(t) have the same meanings as in Equation (2). If we assume that the total population *N* is constant, then the sum of the increase or decrease of the state of each population is 0, namely, $\frac{dS(t)}{dt}+\frac{dI(t)}{dt}+\frac{dR(t)}{dt}+\frac{dD(t)}{dt}=0$.

### 2.2. PINNS for SEIRD Model

**Figure 1:**
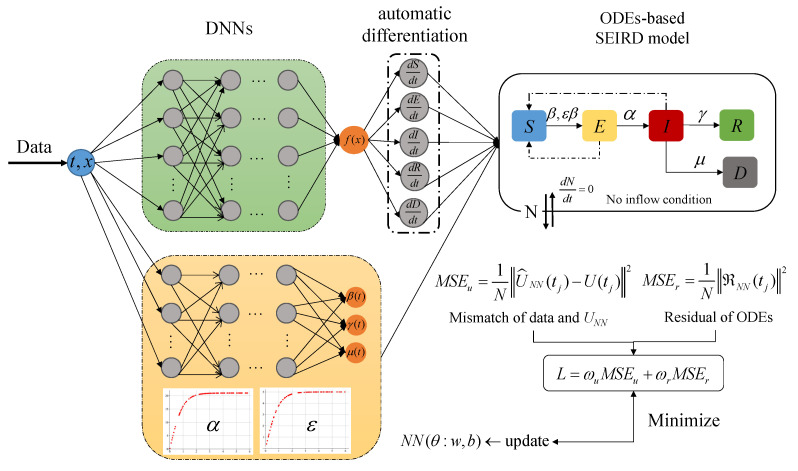
Schematic diagram of the PINNs framework for the SEIRD compartmental model with unknown (time-varying and constant) parameters. The green-shaded DNNs represents the states $U_{\mathrm{NN}}\left(t\right)$ to fit the available data and infer the unobserved dynamics. The yellow-shaded DNNs represents time-varying parameters β(t), γ(t), μ(t). The two constant parameters (α, ϵ) are represented by the modified tanh(t) activation function. The loss comprises two parts: the mismatch between available data and DNNs output and the residual of the SEIRD compartmental model. By minimizing the loss function, the PINNs framework simultaneously fits the data and infers the unobserved dynamics by satisfying the system of ODEs-based SEIRD compartmental model.

Equations in the compartmental model possess coupling and the coefficients are not independent of each other through the lens of biological and epidemics. In this context, we employ two separate DNNs with input *t* to represent the stats U(t) and time-varying parameters, respectively. For the two unknown constant parameters (α, ϵ), we designed the modified tanh activation function to represent them. The expression of the tanh(x) function is $tanh\left(x\right)=\frac{e^{x}-e^{-x}}{e^{x}+e^{-x}}$, and the range of values belong to [−1, 1]. Considering that α>0 and 0≤ϵ≤1, thus we designed the expression of ϵ as tanh(x) function, the expression of α as 21·tanh(x) function, *x* is a random sample with uniform distribution generated from the interval [0, 3]. Meanwhile, COVID-19 transmission involves the analysis of real-world data, for which the available data size tends to be small and sparse. Such a PINNs architecture enables a well-trained model with a limited data set, as shown in Figure 1.

The PINNs framework is required to fit the data and simultaneously satisfy the equations, whereby the loss function includes two parts. The first part is the mismatch between the network output and the available data, and another part is the residual of ODEs. In this study, we employ the approximation $U_{\mathrm{NN}}\left(t;\Theta_{U}\right)\approx U\left(t\right)$ to represent the time-varying SEIRD equations (Equation (3)). The parameters Θ are optimized to achieve the best fit with the observed data. Considering the available data $U_{j}$ at times $t_{1},t_{2},\cdots ,t_{n}$ as training points (ground truth), the mean squared error (*MSE*) is calculated as follows:

(4) $$MSE_{u}=\frac{1}{N}\sum_{j=1}^{N}{\left|{\hat{U}}_{NN}\left(t_{j}\right)-U\left(t_{j}\right)\right|}^{2},$$

Another component of the loss function is the residual of the systems of Equation (1), we define the residual of equations as $R_{NN}\left(t\right)=\frac{dU(t)}{dt}-F\left(U_{\mathrm{NN}},t;\Xi \right)$. The residual, denoted as $R(t;\Theta_{U})$, serves as a metric for assessing the accuracy of the approximation $U_{NN}\left(t;\Theta_{U}\right)$ in satisfying the system of ODEs. Evaluating the residual involves computing the time derivative of the neural network output, which can be accomplished using automatic differentiation [23]. To quantify the discrepancy between the predicted and true solutions, we compute the MSE of the residual:

(5) $$MSE_{r}=\frac{1}{N}\sum_{j=1}^{N}{\left|R_{NN}\left(t_{j}\right)\right|}^{2},$$

where *N* represents the number of data points.

In summary, the loss function of the proposed PINNs method is defined as:

(6) $$L=\omega_{u}MSE_{u}+\omega_{r}MSE_{r}$$

The weight coefficients $\omega_{u}$ and $\omega_{r}$ in the loss function play a crucial role in balancing the optimization process between learning from the data and satisfying the ODEs. These parameters allow fine-tuning of the model’s behaviour and trade-off between the two objectives. By adjusting the values of $\omega_{u},\;\omega_{r}$, the emphasis can be placed on either accurately fitting the available data or ensuring the ODEs constraints are well-satisfied. Consequently, this PINNs model strives to minimize the loss function, effectively learning the underlying physics encoded in the ODEs while accurately capturing the patterns and relationships in the available data.

### 2.3. Neural Network Architecture

Neural networks can be viewed as discretizations of continuous dynamical systems, making them well-suited for dealing with dynamic systems. From a mathematical perspective, the neural networks defines a mapping of the form

(7) $$F:x\in R^{d}⟹y=F\left(x\right)\in R^{c},$$

where *d* and *c* are the input and output dimensions, respectively. Although various types of DNNs have been developed, such as recurrent neural networks, convolutional neural networks, and the well-known Transformers architecture [24,25,26], fully-connected deep neural networks (FDNN) have demonstrated superior performance in scientific computing. Generally, a standard neural unit of an FDNN receives an input $x\in R^{d}$ and produces an output $y\in R^{m}$, y=σ(Wx+b) with $W\in R^{m\times d}$ and $b\in R^{m}$ being weight matrix and bias vector, respectively. σ(·) referred to as the activation function, is designed to add element-wise non-linearity to the model. An FDNN with *ℓ* hidden layers can be considered a nested composition of sequential standard neural units. Specifically, the $j_{th}$ neuron in *ℓ* layer can be formulated as

(8) $$y_{j}^{\left[\ell \right]}=\sum_{k=1}^{n^{\left[\ell -1\right]}}w_{jk}^{\left[\ell \right]}\sigma^{\left[\ell -1\right]}\left(y_{k}^{\left[\ell -1\right]}\right)+b_{j}^{\left[\ell \right]},$$

where $y_{k}^{\left[\ell -1\right]}$ represents the value of the $k_{th}$ neuron in the ℓ−1 layer, $n^{\left[\ell -1\right]}$ represents the number of neurons in the ℓ−1 layer, $\sigma^{\left[\ell -1\right]}$ is the activation function of the ℓ−1 layer, $w_{jk}^{\left[\ell \right]}$ is the weight between the $k_{th}$ neuron in the ℓ−1 layer and the $j_{th}$ neuron in the *ℓ* layer, and $b_{j}^{\left[\ell \right]}$ is the bias of the $j_{th}$ neuron in the *ℓ* layer.

Residual Network architecture (ResNet) was proposed as a solution to the problem of vanishing/exploding gradients in DNNs in various computer vision tasks [27]. ResNet introduces skip connections, allowing gradient flow through alternate shortcut paths. This enables the model to learn identity functions, ensuring that higher layers perform at least as well as lower layers [28,29]. Considering the aforementioned benefits, we adopt a one-step skip connection strategy in the FDNN architecture, connecting two consecutive layers that have an equal number of neurons. However, if the consecutive layers have different numbers of neurons, the skip connection step is omitted. Mathematically, a ResNet block with a one-step connection produces a filtered version $y^{\left[\ell +1\right]}\left(x;\theta \right)$ for the input $y^{\left[\ell \right]}\left(x;\theta \right)$ as follows:

(9) $$y^{\left[\ell +1\right]}\left(x;\theta \right)=y^{\left[\ell \right]}\left(x;\theta \right)+\sigma \circ \left(W^{\left[\ell +1\right]}y^{\left[\ell \right]}\left(x;\theta \right)+b^{\left[\ell +1\right]}\right).$$

For solving differential equations, the first and second derivatives of the neural networks would serve as inputs to calculate the loss function, which means that the activation function of the DNNs in the PINNs framework requires the second derivative to be satisfied as non-zero. Therefore, the activation function has an extremely significant impact on the success of training PINNs. Many research works have demonstrated that the *sigmoid* function and *tanh* function are suited for effective PINNs framework training tasks. In this study, we selected the *tanh* function as the activation function for each layer.

## 3. Numerical Simulations

### 3.1. Data and Settings

#### 3.1.1. Data

**Figure 2:**
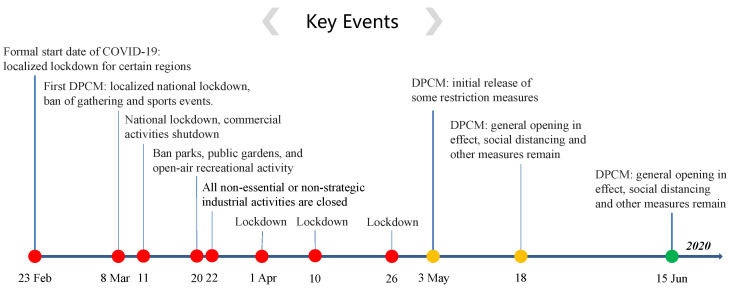
Timeline of NPIs implemented in Italy to control COVID-19. DPCM: Decree of the Prime Minister.

For the COVID-19 epidemic in Italy, the first official report of indigenous case was on 21 February 2020 in Lodi province, while several epidemiological-linked cases were traced back to 20 February 2020. The data considered in our study is downloaded from Italian Civil Protection (http://www.protezionecivile.gov.it/media-comunicazione/comunicati-stampa) and Ministry of Health (http://www.salute.gov.it/portale/home.html). It is comprised of commutative infected, recovered, and deceased cases for the period from 20 February 2020 (day 1), to 30 June 2020 (day 132) [30]. In order to control the transmission of COVID-19 in Italy, lockdown and many NPIs were implemented from 23 February 2020, as the developed timeline shown in Figure 2. All events and interventions are available from official websites (https://mn.gov/governor/covid-19/news/). To avoid weekly fluctuations induced by the work-leisure shift and nature noise in the real-world data, a 7-day moving average was used to smooth the reported data by averaging the values of each day with those of the 7 days before.

#### 3.1.2. Settings

We implement the PINNs method using Python and the PyTorch framework [31]. Each neural networks implemented in this paper comprise 5 layers, where the weight matrix $W_{k}$ and the bias vector $b_{k}$ of the $k^{th}$ layer are respectively $W_{1}\in R^{1\times 35}$, $W_{2}\in R^{35\times 50}$, $W_{3}\in R^{50\times 30}$, $W_{4}\in R^{30\times 30}$, $W_{5}\in R^{30\times 20}$ and $b_{1}\in R^{35}$, $b_{2}\in R^{50}$, $b_{3}\in R^{30}$, $b_{4}\in R^{30}$, $b_{5}\in R^{20}$. For the numerical experiment, we train the neural networks using the Adam optimizer with an initial learning rate of $2\times 10^{-3}$ and a decay rate of 95% every 2000 epochs. The entire training process takes about 10 min to run 50,000 epochs on all training data, and predictions can be made within seconds.

### 3.2. Fitting and Predictions

**Figure 3:**
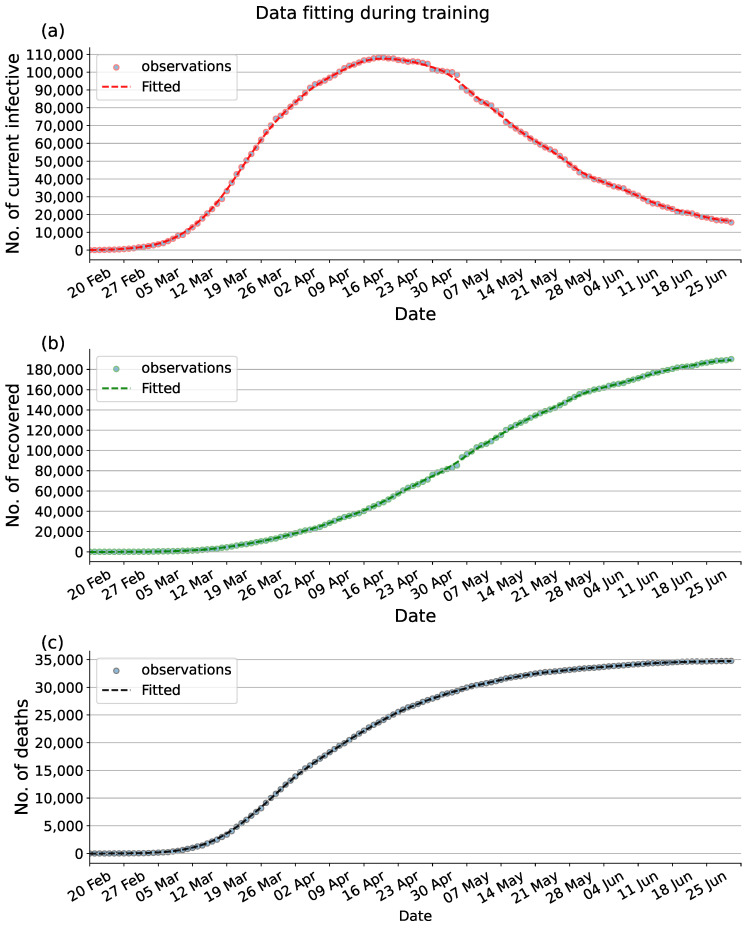
Data fitting during training. (**a**) Fitting to the available data of current infectious. (**b**) Fitting to the available data of cumulative recovered. (**c**) Fitting to the available data of cumulative deaths. Dot: observed data. Dashed: Fitted data.

**Figure 4:**
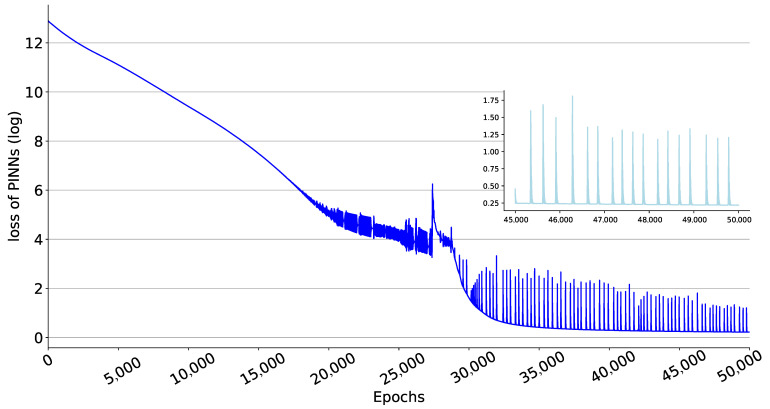
Loss of PINNs during the training process. The value of the Equation (6) is taken as log. The subplot shows the loss of the last 5000 epochs of training, with all loss values less than 2, which is a minimal loss compared to the range of values of the ODEs.

Through the compartmental model to analyze historical data, epidemiological parameters, and predict future trends of the epidemic, the modelling results can provide reliable feedback information for the authorities to make future decisions. To assess the fitting performance of the proposed PINNs method with the observed data between 20 February 2020, and 30 June 2020, we visualize the data from the final epoch of training in Figure 3. Figure 3a represents the number of current infections, Figure 3b shows the cumulative number of recoveries, Figure 3c displays the cumulative number of deaths. In addition, as can be seen in Figure 4, the value of the loss function guarantees the convergence of the proposal PINNs method for the SEIRD compartmental model.

**Figure 5:**
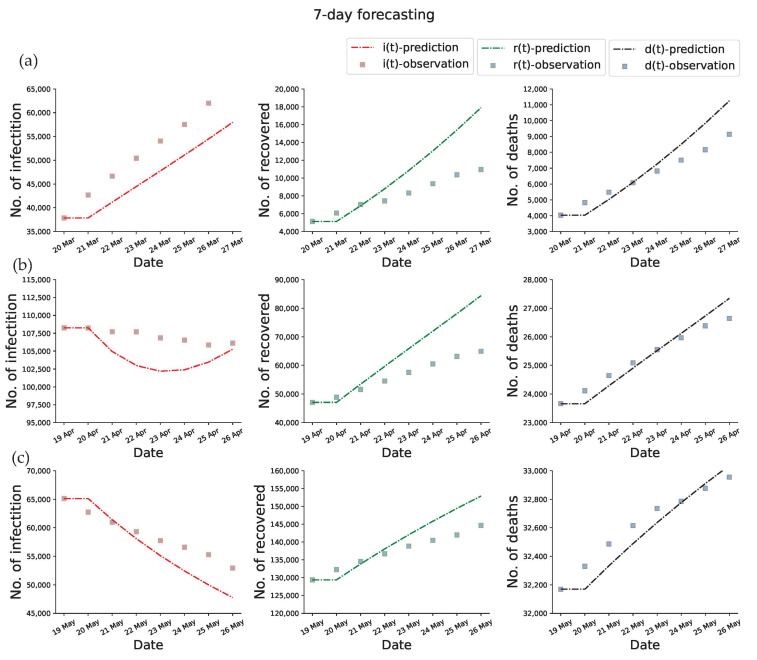
Fitting and 7-day prediction of PINNs for SEIRD model. The first column is plotted the predicted current infections, the second column is plotted the predicted cumulative recovered, the third column is plotted the predicted cumulative deaths, and the dotted boxes represent the corresponding observations. (**a**) 7-day forecasting results based on the 20 February to 20 March 2020 time window. (**b**) 7-day forecasting results based on the 20 February to 19 April 2020 time window. (**c**) 7-day forecasting results based on the 20 February to 19 May 2020 time window.

We tested the forecasting power of the proposed method by performing predictions for the early outbreak of COVID-19 in Italy at one-month, two-month, and three-month, respectively. The ODEs-based compartmental model requires determined initial conditions and model parameters to make predictions. As the initial conditions can be obtained from the training data and the model parameters are already calibrated, we can forecast the epidemic dynamics by performing the forward process. In the prediction part, the value of β(t), γ(t), and μ(t) are assumed to be their final value of the training time window. Figure 5 displays the data fitting and prediction obtained by using the proposed PINNs method for the SEIRD model. Figure 5a displays the one-week prediction based on the reported data from 20 February to 20 March 2020, Figure 5b displays the one-week prediction based on the reported data from 20 February to 19 April 2020, and Figure 5c displays the one-week prediction based on the reported data from 20 February to 19 May 2020. The perfect match between the predictions and the observations demonstrates the parameters inferred by the learned network are very plausible, as well as the generalization ability of the model. Note that the number of recovered and death states in the SEIRD model are terminal states, which means that the changes in the number of recovered and death people are always non-decreasing. In turn, the infected people may see periods of increase and decrease due to it being a state of transition.

Moreover, to quantitatively assess the performance of the proposed PINNs method, we utilize three evaluation metrics for fair and effective comparisons: mean absolute error (*MAE*), root mean square error (*RMSE*), and mean absolute percentage error (*MAPE*). The calculation method is illustrated in Equations (10)–(12).

(10) $$MAE=\frac{1}{n}\sum_{i=1}^{n}\left|\hat{y_{i}}-y_{i}\right|,$$

(11) $$RMSE=\sqrt{\frac{1}{n}\sum_{i=1}^{n}{\left(\hat{y_{i}}-y_{i}\right)}^{2}},$$

(12) $$MAPE=\frac{1}{n}\sum_{i=1}^{n}\frac{|\hat{y_{i}}-y_{i}|}{\hat{y_{i}}}*100\%,$$

Experimental results as represented in Table 1 show the highly accurate forecasting capability of the proposed method.

### 3.3. Inference

**Figure 6:**
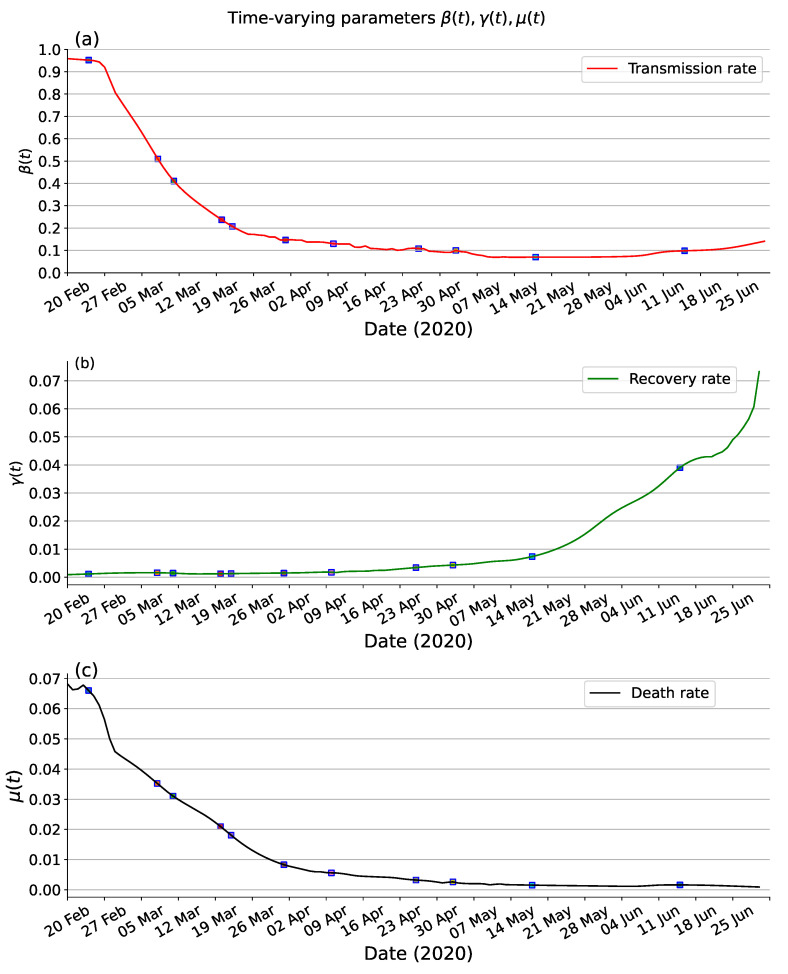
The time-varying transmission rate of SEIRD model based on PINNs method on Italy data from 20 February to 30 June 2020. (**a**) transmission rate β(t). (**b**) recovery rate γ(t). (**c**) death rate μ(t).

Estimating the unknown parameters in compartmental models is crucial for understanding the dynamics of disease transmission and evaluating the effectiveness of interventions. These parameters, including transmission rates, incubation period, recovery rate and mortality rate play a pivotal role in shaping the spread of infectious diseases. The incubation period and the infectiousness during this period are parameters specific to the virus, which can be obtained from clinical case information or inferred using statistical or mathematical modelling based on available data. The estimated incubation period of COVID-19 is approximately 5.8 days, with the infectiousness during this period found to be nearly equal to 99.9% of the infection period. Figure 6 presents the estimated time-varying parameters in Italy from 20 February to 30 June 2020. This analysis provides insights into how the values of β(t), γ(t), and μ(t) change over the specified period, reflecting the impact of interventions and other factors on the dynamics of the disease. As shown in Figure 6a, the transmission rate β(t) can fit well with what would be expected given such events. The earliest traceable first confirmed case of COVID-19 on 20 February 2020, the authorities of Italy started imposing a localized lockdown for certain regions on 23 February 2020, these control measures achieved a certain success, as demonstrated by a significant reduction in transmission rates β(t). As far as γ(t) and μ(t), hospitals’ ability particularly emergency rooms had a considerable impact. In the context of COVID-19, hospitals are at full capacity in the first months of the outbreak, and as months went by, healthcare professionals learned more about possible treatments to treat the disease’s symptoms and effects. This usually results in a decrease in the proportion of individuals that died from the disease (decrease of μ(t)) and in a decrease in the recovery time (an increase of γ(t)). As shown in Figure 6b,c, in qualitative terms, was an increasing trend in γ(t) and a decreasing trend in μ(t).

**Figure 7:**
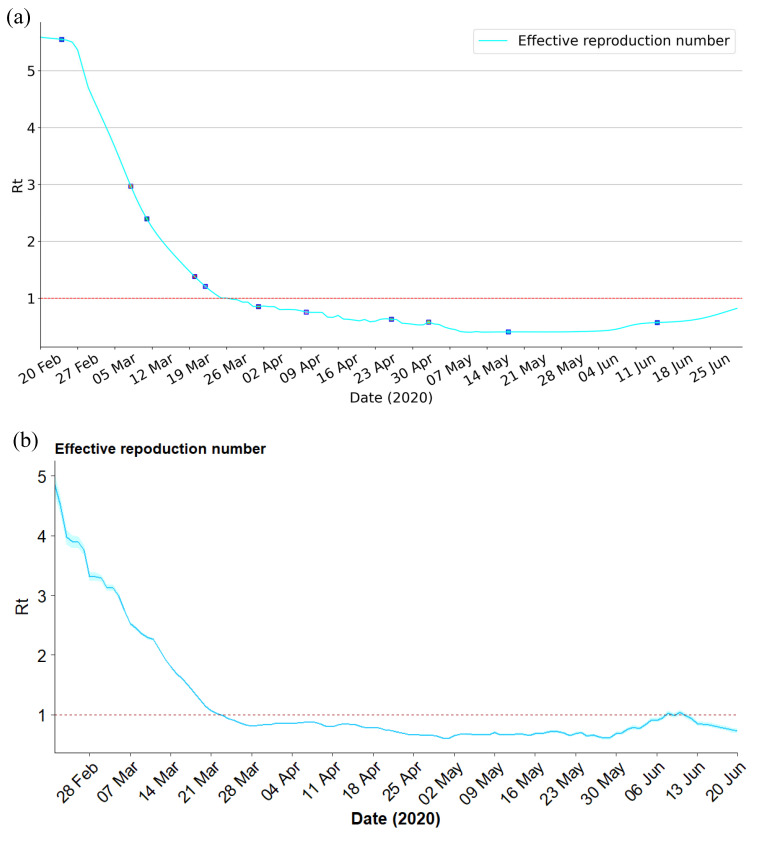
$R_{t}$ in Italy from 20 February to 30 June 2020. (**a**) Rt estimated by proposed PINNs method for SEIRD model. (**b**) $R_{t}$ estimated by serial Bayesian model.

Effective reproduction number $R_{t}$ is a crucial parameter, $R_{t}$ less than 1 indicates that the transmission of the infectious disease will gradually disappear. By monitoring changes in $R_{t}$ over time, public health officials can make informed decisions about interventions to control the spread of the disease. $R_{t}$ can be calculated by the Next Generation Matrix (NGM) approach [32], for the given SEIRD model (3), $R_{t}=\epsilon \cdot \beta \left(t\right)\alpha +\frac{\beta (t)}{\gamma (t)+\mu (t)}$. Figure 7a illustrates the evolution of $R_{t}$ of the SEIRD compartmental model from 20 February to 30 June 2020, in Italy. In the first several days of the outbreak, the effective reproduction number $R_{t}$ was greater than 5, which resulted in a substantial outbreak. On 25 February, $R_{t}$ gradually decreased as localized lockdown for certain regions and the awareness of the epidemic. However, $R_{t}$ was still greater than 1, which may be due to the partially incomplete lockdown, or the movement of people from northern to southern Italy when the country-wide lockdown was announced but not yet enforced. When the national lockdown was fully operational and strictly enforced, $R_{t}$ keeps decreasing and finally reached below 1. Moreover, $R_{t}$ steadily declined at the end of March due to a wider testing campaign that identified more mildly symptomatic infected individuals. Since 15 June, $R_{t}$ shows a growing trend due to DPCM declaring that general opening was in effect, social distancing, and other measures remained. Additionally, to validate the estimated $R_{t}$, a serial Bayesian model was implemented to produce the $R_{t}$ of Italy at the same time period [33], as shown in Figure 7b. Parameters for the serial interval distribution in the model were set according to the published literature (mean = 7.5 d; SD = 3.4 d) [34]. As we can see, the $R_{t}$ estimated by the proposed PINNs method is essentially the same as that estimated by the Bayesian model.

## 4. Discussion

Transmission modelling is increasingly being used to support public health decision-making in the control of infectious diseases. In this study, we introduce a modified SEIRD compartmental model with time-varying parameters to analyze and forecast the transmission dynamics of COVID-19 in Italy. Figure 6a reveals the efficacy of intervention measures imposed by the authorities in reducing the key transmission rate parameter β(t). Figure 6b,c show that the recovery rate tends to increase over time and the death rate to decrease. These trends may not be directly related to the lockdown but could be attributed to various factors, among which a better understanding of the disease and consequent improvement in the effusiveness of the response from the national health system, and possibly a change in the nature, virulence, and lethality of the virus.

Moreover, we evaluate the goodness of fit of the estimated parameters fit the SEIRD compartmental model by comparing our results with those of previous publications. We compare our results to those obtained using the method of the rolling regression framework [35], where the order of magnitude of the time-varying parameters β(t), γ(t), and μ(t) is in agreement and the trend is almost identical. A comprehensive meta-analysis demonstrated that the median incubation period for general transmissions in early outbreaks was 5.8 days (95% confidence interval: 5.3 to 6.2) [36]. Li et al. analyzed data on the first 425 confirmed cases in Wuhan to determine the epidemiologic characteristics of novel coronavirus infected pneumonia, the results show that the mean incubation period was 5.2 days (95% confidence interval: 4.1 to 7.0) [37]. Yang et al. collected contact tracing data in a municipality in Hubei province during a full outbreak period to estimate the incubation period and serial interval of COVID-19, the estimated median incubation period of COVID-19 is 5.4 days (95% confidence interval: 4.8 to 6.0) [38]. The estimated α by the proposed PINNs method is 5.8, which is consistent with the results of the above research. The estimated ϵ by the proposed PINNs method is 0.99, which means that the transmission capacity of exposed and onset populations are nearly identical [39]. Numerous related studies demonstrate that the incubation period and the infection period carry almost the same capacity for transmission [40,41]. Findings demonstrate that the proposed PINNs method yields reliable results that are consistent with the expected dynamics and the results of previous publications [42].

We have presented a comprehensive workflow for analyzing infectious disease transmission systems described by a system of ODEs produced compartmental model. It is noteworthy that our proposed method, based on PINNs, requires minimal knowledge of numerical analysis, such as stability conditions, but relies on familiarity with neural network libraries. The versatility of the PINNs method allows effective simulation of various epidemic scenarios, hypothesis testing, and the design of appropriate control measures within the given scenario under consideration. While the proposed PINNs method indeed offers many advantages, it does have some limitations. One of the main limitations is that PINNs architecture requires prior knowledge of the physical laws and constraints that govern the problem being solved. The structure of compartmental models may change depending on the question of interest and impact their accuracy. That means if the underlying epidemiological laws are not well understood or if the available data is not consistent with the known epidemiological laws, the model may not work well. But it should be noted that the emphasis on infectious disease models is on the application of public health, not the mathematics of these models. As world-renowned Statistician George E. P. Box made the following statement. “All models are wrong, but some are useful”.

## 5. Conclusions

We developed a PINNs method to estimate both time-varying and constant parameters for an ODE-based compartmental model of COVID-19 transmission. By applying this method to model the early outbreak of COVID-19 in Italy, we achieved effective fitting of the contagion data and accurate predictions of its evolution. The results with real-world data demonstrate that our proposed model accurately captures the real-time dynamics of the contagion, offering reliable predictions and valuable insights into the mechanisms of transmission.

## Figures and Tables

**Table 1:** The forecasting performance in 3-day, 5-day, and 7-day.

Metrics	After 20 March 2020			After 19 April 2020			After 19 May 2020		
	3-Day	5-Day	7-Day	3-Day	5-Day	7-Day	3-Day	5-Day	7-Day
MAE(I)	5411	5790	6419	2503	3258	2792	1352	2170	3046
RMSE(I)	5431	5819	6519	3705	2618	3275	1567	2515	3514
MAPE(I)	11.60%	11.52%	11.78%	2.32%	3.04%	2.61%	2.20%	3.70%	5.41%
MAE(R)	813	1728	2944	2934	5704	9001	1643	2700	4170
RMSE(R)	959	2128	3706	3321	6821	10,936	1880	3151	4972
MAPE(R)	11.93%	20.07%	31.04%	5.57%	10.00%	14.83%	1.23%	1.96%	2.97%
MAE(D)	423	543	927	330	235	318	147	109	95
RMSE(D)	527	637	1151	349	279	379	147	122	109
MAPE(D)	8.36%	8.98%	12.64%	1.35%	0.95%	1.24%	0.45%	0.34%	0.30%

## Data Availability

All data is available.
